# Conditional Tek Promoter-Driven Deletion of Arginyltransferase in the Germ Line Causes Defects in Gametogenesis and Early Embryonic Lethality in Mice

**DOI:** 10.1371/journal.pone.0007734

**Published:** 2009-11-05

**Authors:** Nicolae Adrian Leu, Satoshi Kurosaka, Anna Kashina

**Affiliations:** Department of Animal Biology, School of Veterinary Medicine, University of Pennsylvania, Philadelphia, Pennsylvania, United States of America; University of Texas MD Anderson Cancer Center, United States of America

## Abstract

Posttranslational protein arginylation mediated by Ate1 is essential for cardiovascular development, actin cytoskeleton functioning, and cell migration. Ate1 plays a role in the regulation of cytoskeleton and is essential for cardiovascular development and angiogenesis—capillary remodeling driven by in-tissue migration of endothelial cells. To address the role of Ate1 in cytoskeleton-dependent processes and endothelial cell function during development, we produced a conditional mouse knockout with Ate1 deletion driven by Tek endothelial receptor tyrosine kinase promoter expressed in the endothelium and in the germ line. Contrary to expectations, Tek-Ate1 mice were viable and had no visible angiogenesis-related phenotypes; however, these mice showed reproductive defects, with high rates of embryonic lethality in the second generation, at stages much earlier than the complete Ate1 knockout strain. While some of the early lethality originated from the subpopulation of embryos with homozygous Tek-Cre transgene—a problem that has not previously been reported for this commercial mouse strain—a distinct subpopulation of embryos had lethality at early post-implantation stages that could be explained only by a previously unknown defect in gametogenesis originating from Tek-driven Ate1 deletion in premeiotic germs cells. These results demonstrate a novel role of Ate1 in germ cell development.

## Introduction

Protein arginylation is a posttranslational modification that constitutes addition of arginine to proteins and is mediated by arginyltransferase (Ate1) [1]–[3]. Mice lacking Ate1 die between embryonic days E12.5 and E17.5 with severe cardiovascular defects, including abnormal heart development and angiogenesis [4], [5]. While Ate1 knockout (KO) embryos initially develop normal blood vessels in the process of vasculogenesis, the capillary network formation during subsequent angiogenesis is impaired, leading to defective capillary branching and their premature termination. These defects result in paleness, thin blood vessels, frequent skin edemas, and hemorrhages in the Ate1 KO embryos, and have been previously proposed to underlie the lethality in Ate1 KO mice, however the mechanisms of these defects, and the cells and tissues responsible for the Ate1−/− angiogenic phenotypes, are unknown.

Past work from our lab showed that a prominent subset of proteins arginylated in vivo constitutes structural and functional components of the cytoskeleton [6], with roles in cell migration and cell division – the processes that are important during many developmental stages from gametogenesis to organ morphogenesis. Ate1 KO results in impaired cell motility that originates from actin cytoskeleton defects at the cell leading edge [7]. It has also been established by multiple groups that embryonic angiogenesis is driven by endothelial cells that migrate through tissues in the developing organism, forming branches off the existing blood vessels and laying out the mature circulatory system (reviewed in [8], [9]). Taken together, these findings lead to a hypothesis that Ate1-dependent regulation of angiogenesis occurs through regulation of the motility of endothelial cells during tissue remodeling in embryogenesis, and that Ate1 function may also be essential for other cytoskeleton-dependent processes in development. To test this hypothesis, we generated a conditional knockout model (Tek-Ate1), in which Ate1 deletion is driven by the mouse Tek promoter of endothelial receptor tyrosine kinase (also known as Tie2) that is expressed in endothelial cells and the germ line, and studied the phenotype of the resulting Tek-Ate1 mice.

Contrary to our expectations, Tek-Ate1 mice were viable and had no visible angiogenesis-related phenotypes, suggesting that Ate1 plays no major role in endothelial cells during development. At the same time, these mice showed reproductive defects, with high rates of embryonic lethality in the second generation, at stages much earlier than the complete Ate1 KO strain. Control matings showed that some of the early lethality originated from the subpopulation of embryos with homozygous Tek-Cre transgene, a problem that has not previously been reported for this commercial mouse strain. However, we also found a distinct subpopulation of embryos whose lethality at early post-implantation stages could be explained only by a previously unknown defect in gametogenesis originating from Tek-driven Ate1 deletion in premeiotic germs cells. Further analysis showed that the combined defects of Tek-Ate1 knockout lead to an overall ∼80% embryonic lethality. These results demonstrate a previously unknown role of Ate1 in germ cell development.

## Results

### Tek-Ate1 conditional knockout (CKO) mice exhibit high rates of embryonic lethality in the second generation

To address the role of Ate1 deletion in the endothelial cells, we generated a conditional Tek-Ate1 knockout mouse line (CKO), by crossing the Ate1-floxed mice, in which the first three exons of the Ate1 gene are flanked by LoxP sites, with a commercially available mouse line, in which expression of Cre recombinase is driven by Tek (Tie2) promoter [10] (Figure S1). In this knockout, the expected Ate1 deletion affects the critical region of the Ate1 gene, previously shown to be responsible for Ate1 activity [2], [11]. Using R26R Rosa reporter mouse strain, it was confirmed that the expression of the transgene occurs in the embryos as expected (Figure S2). The offspring of Ate1-floxed and commercially obtained Tek-Cre mice (Tek floxed/+) were used as founder animals in the present study.

**Figure 1:**
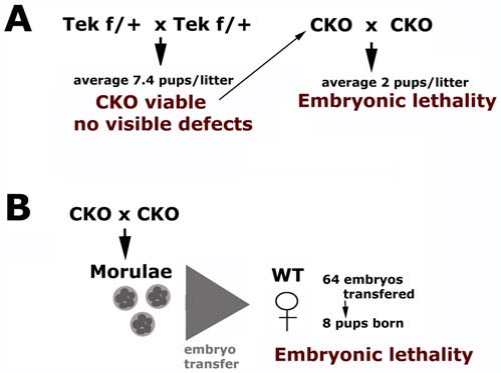
Tek-Ate1 CKO mice exhibit high rates of embryonic lethality in the second generation. (A) Left: In the first generation, mating of Tek floxed/+ (Tek f/+) parents produces normal size litters (numbers shown are calculated based on 52 pups born from 7 pregnant females); CKO mice in these litters (identified by Tek floxed/floxed genotype as shown in Figure S1) are viable with no visible defects. Right: Mating of CKO mice to produce second generation of CKO pups leads to high rates of embryonic lethality, with an average of only two liveborn pups per litter (numbers shown are calculated based on 8 pups born from 4 pregnant females). (B) Embryos from CKO × CKO matings were collected at the morula stage and transferred into wild type recipient females. Only 8 pups were born from 64 transferred morulae, confirming that the embryos in CKO × CKO matings have high rate of embryonic lethality.

The first generation of CKO mice was successfully obtained by the mating of Tek floxed/+ parents (Figure 1). These matings produced fairly normal litter sizes (average 7.4 pups/litter), and the CKO mice from these litters (Tek floxed/floxed genotype) were viable, without any visible defects (Figure 1A left). However, when these CKO females and males were mated to produce the second generation, only 2 pups per litter were obtained, indicating high rates of embryonic lethality in the second generation (Figure 1A right). To test the possibility that this embryonic lethality was due to the inability of CKO females to carry a healthy pregnancy to term (due to angiogenic defects that could interfere with normal blood supply to the fetuses), we transferred pre-implantation embryos from CKO × CKO matings into wild type females and analyzed the lethality of the resulting progeny. These embryo transfers resulted in similar lethality rates to those observed in the matings: out of 64 embryos transferred, only 8 (i.e., 12.5%) developed to term. Therefore, the lethality observed in CKO matings was due to the defects in the embryos (Figure 1B).

### Progeny of Tek-Ate1 CKO mice have delayed development and die at early post-implantation stages

**Figure 2:**
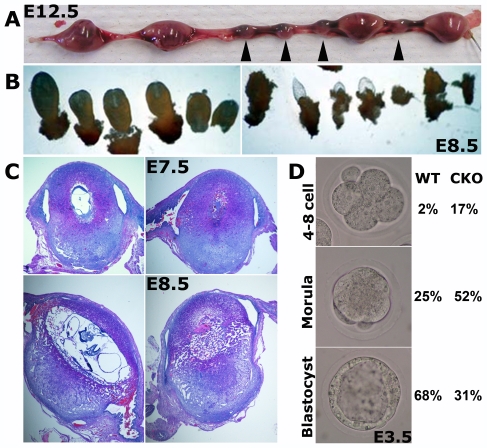
Tek-Ate1 CKO mice have delayed development and die at early post-implantation stages. (A) Uterus excised from pregnant CKO female mated to CKO male at 12.5 day post coitum (12.5 dpc); arrowheads indicate the embryos that have implanted but either died or were severely delayed in development compared to their littermates. (B) Individual embryos excised from E8.5 uterus derived from CKO × CKO mating. Left panel shows the embryos that appeared normal in size and morphology. Right panel shows the embryos from the same uterus that appeared dead and partially deteriorated at E8.5. (C) Cross section through the uteri derived from CKO females mated to CKO males, showing individual littermate embryos at E7.5 (top) and E8.5 (bottom). While approximately half of the embryos at each stage appeared normal (left panels), the other half of the embryos in the same uterus were severely deteriorated (right panels), suggesting that these embryos have been dead by the time of dissection. (D) Left, examples of individual embryos at 4–8 cell stage (top), morula (middle), and blastocyst (bottom), derived from pregnant females at E2.5 and cultured for 1 day in vitro. Percentages in the columns on the right are calculated from the numbers of embryos found as 4–8 cell, morula, and blastocyst stage as marked on the left, derived from Ate1-floxed (WT) and CKO matings.

According to the supplier, in addition to endothelial cells Tek promoter is also activated in hematopoetic cells and in the germ line, leading to a possibility that CKO mice in the second generation have Tek-Cre-driven homozygous deletion of Ate1 coming from the germ line, and are in effect identical to the previously characterized Ate1−/− mice, lethal at E12.5–E17.5 [4]. We tested this possibility by euthanizing the pregnant CKO females 12.5 days post coitum (dpc) and observing the numbers and morphology of the embryos. Surprisingly, we found that nearly half of the E12.5 embryos in each litter were of an abnormally small size (Figure 2A), suggesting that these embryos were severely delayed or dead long before E12.5. Such a phenotype was never observed in the complete Ate1 knockout.

To find whether the decreased embryo size observed at E12.5 reflects lethality or developmental delay, we euthanized pregnant CKO females at 8.5 dpc and excised individual embryos from the uteri to observe their morphology. We found that approximately half of the embryos at this stage appeared normal (Figure 2B, left panel), while the other half was severely deteriorated (Figure 2B, right panel), suggesting that these embryos have been dead by the time of dissection. Sectioning of the uteri from pregnant CKO females at 7.5 and 8.5 dpc (Figure 2C) suggested that even at E7.5 a large percentage of the embryos were dead (Figure 2C, top right) and indicated that the lethality observed in the second generation CKO mice occurs at early post-implantation stages.

To test whether this lethality correlates with any early developmental defects that can be seen at pre-implantation stages, embryos from CKO × CKO matings were collected at E2.5 (morula stage) and cultured in vitro for 1 day to reach E3.5 (blastocyst stage). Since it is known that in such experiments the developmental rate differs for different mouse strains, two strains were used as a control: B6C3 (a strain known for its good in vitro development) and Ate1-floxed (a mouse strain that matches the CKO in the strain background and handling, while being, for all intents and purposes, a wild type strain). In B6C3 mice (not shown) all embryos reached blastocyst stage by E3.5 in culture (n = 40). The development of Ate1-floxed mice was slower and more variable, with E3.5 embryos found at blastocyst, morula, and 4–8 cell stages at 68%, 25% and 2%, respectively (Figure 2D, n = 47). In comparison, the development of the CKO × CKO was significantly delayed, with only a minority of embryos reaching the blastocyst stage by E3.5 and a significant percentage of embryos having abnormal appearance and being delayed at 4–8 cell stage even at E3.5. The percentages of blastocysts, morulae and 4–8 cell stage CKO embryos at E3.5 were 31%, 52% and 17%, respectively (Figure 2D, n = 29). Therefore, the developmental delay in CKO × CKO starts at the pre-implantation stages.

### CKO females do not have angiogenic defects that affect pregnancy

**Figure 3:**
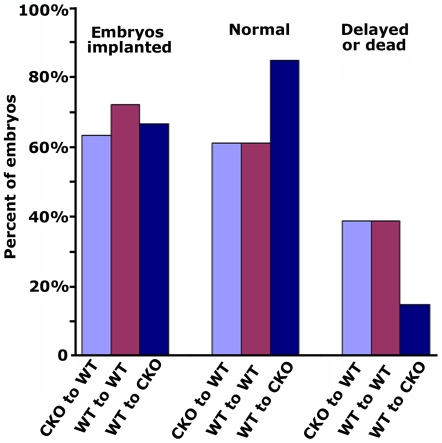
CKO females do not have angiogenic defects that affect pregnancy. Embryos from CKO × CKO matings were transferred into WT female recipients (CKO to WT) and vice versa (WT to CKO); as a control, WT embryos were transferred to WT females (WT to WT). Recipient females were euthanized at E10.5–E12.5 and the percentages of embryos were scored as ‘embryos implanted’ (i.e., implanted in the uterus to produce a visible decidua, left set of bars), ‘normal’ (i.e., reaching expected size by E12.5, middle set of bars), and ‘delayed or dead’ (i.e., small and partially deteriorated by the time of the euthanasia, right set of bars). Percentages shown in the figure were derived from the following embryo numbers: for CKO to WT, out of 93 embryos transferred, 59 were recovered at E12.5 (shown as ‘embryos implanted’), including 36 normal and 23 delayed or dead; for WT to WT, out of 75 embryos transferred, 54 were recovered, including 35 normal and 19 delayed or dead; for WT to CKO, out of 30 embryos transferred, 20 were recovered, including 17 normal and 3 delayed or dead.

The data described above show that second generation CKO embryos have developmental defects and high rates of early embryonic lethality, however, it is still possible that defects in the CKO females also contribute to the early lethality observed in these mice. Indeed, if CKO mice have endothelial-driven angiogenic defects, such defects may result in a decreased blood supply to the fetus and uterine incompetence and could lead to delayed development and death at early post-implantation stages. In addition, these mice may also exhibit defects in wound healing that heavily rely on the angionenic processes. To test these possibilities, we performed embryo transfer experiments, in which control embryos were transferred into CKO females and vice versa (WT to CKO and CKO to WT, respectively, as shown in Figure 3), followed by euthanasia and uteri removal at mid-development to observe the size and morphology of the embryos. We found that CKO females were as capable as WT to recover from the surgery preformed during embryo transfer (indicating their normal wound healing abilities), as well as to support pregnancy (80% of transferred embryos implanted and reached normal size at E8.5, compared to 60–65% in the control females) (Figure 3). This result and the data shown in Figure 1 indicate that CKO females have normal angiogenic-related processes, can support embryonic development until E8.5, and that the embryonic death and developmental delays shown in Figure 2 are due exclusively to the embryo-specific defects.

### Early lethality in Tek-Ate1 CKO mice is due to a combination of Tek-dependent lethality and Ate1-specific non-angiogenic defects

**Figure 4:**
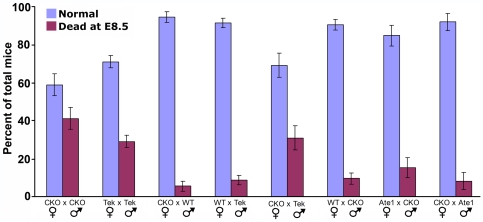
Early lethality in Tek-Ate1 CKO mice is due to a combination of Tek-dependent lethality and Ate1-specific non-angiogenic defects. Females and males of different genotypes were mated in the combinations shown in the figure, and the percentages of normal and dead embryos in each litter were calculated by euthanizing the pregnant females at mid-development (on or around E12.5), excising the uteri, and scoring the numbers of normal and dead embryos as described in the text and legend to Figure 3. Numbers used to derive percentages are shown in the text. Error bars represent SEM. For the comparison between CKO × CKO and TekxTek, the t-value is 1.84, the degree of freedom is 277, and the p-value (one sided tail) is 0.033—using the Welch t-test method for two distributions with different means and variances.

The data described above suggest that Tek promoter-driven Ate1 deletion results in early embryonic lethality that does not depend on the functions of the endothelial cells. To determine the possible factors that may contribute to such lethality, we performed a series of control matings using CKO mice, Tek-Cre line, and the original Ate1 +/− line [5] (Figure 4). In each mating, pregnant females were euthanized at ∼12.5 dpc and the numbers of embryos that appeared abnormally small and/or partially deteriorated at this stage (as shown in Figure 2A) were scored as ‘dead at E8.5’ to produce the bar diagram shown in Figure 4.

41.1% of fetuses in CKO × CKO matings appeared dead at E8.5 (n = 73), confirming our earlier observations that nearly half of the embryos have severe developmental problems at this stage. However, in control Tek-Cre x Tek-Cre and CKO x Tek-Cre matings, the rates of the lethality at E8.5 were 29.0% (n = 207) and 31.0% (n = 55), respectively, suggesting that the lethality was high when both parents had Tek-Cre allele, regardless of Ate1 deletion. Matings in other combinations shown in Figure 4 showed that in most cases these strains of mice also exhibited a ‘baseline’ ∼5–10% lethality rate (0–2 dead embryos per litter in different matings), suggesting that some embryos die without any genetic defects. Specifically, the lethality in matings of WT females × Tek-Cre males was 8.6% (n = 128), CKO females × WT males 5.5% (n = 73), WT females × CKO males 9.5% (n = 105), CKO females × Ate1+/− males 8.1% (n = 37), and Ate1+/− females × CKO males 14.9% (n = 47). Further calculations and genotyping of the surviving progeny (data not shown) suggest that homozygous Tek-Cre results in complete embryonic lethality before E8.5 (∼25% of the progeny). While this lethality rate partially masks the lethality observed in Tek-Ate1, the overall rates of early lethality of the CKO embryos suggest that on top of the homozygous Tek-Cre, CKO mice have an additional reason for lethality, related to Ate1 deletion in tissues other than endothelial cells.

### Ate1-dependent early lethality in Tek-Ate1 mice originates from defects in gametogenesis

As mentioned above, in addition to endothelial cells, leaky Tek-driven Cre expression occurs in hematopoietic cells and the germ line, resulting in the deletion of the loxP-franked alleles in these tissues with unspecified rates. However, since the effect of Ate1 on hematopoetic cells is expected to manifest itself later in development, Ate1 deletion in the hematopoetic cells of the embryos is unlikely to produce the lethality at or before E7.5 as seen in our study. Therefore, it appears likely that the lethality observed here is due to Ate1 deletion in the germ line.

**Figure 5:**
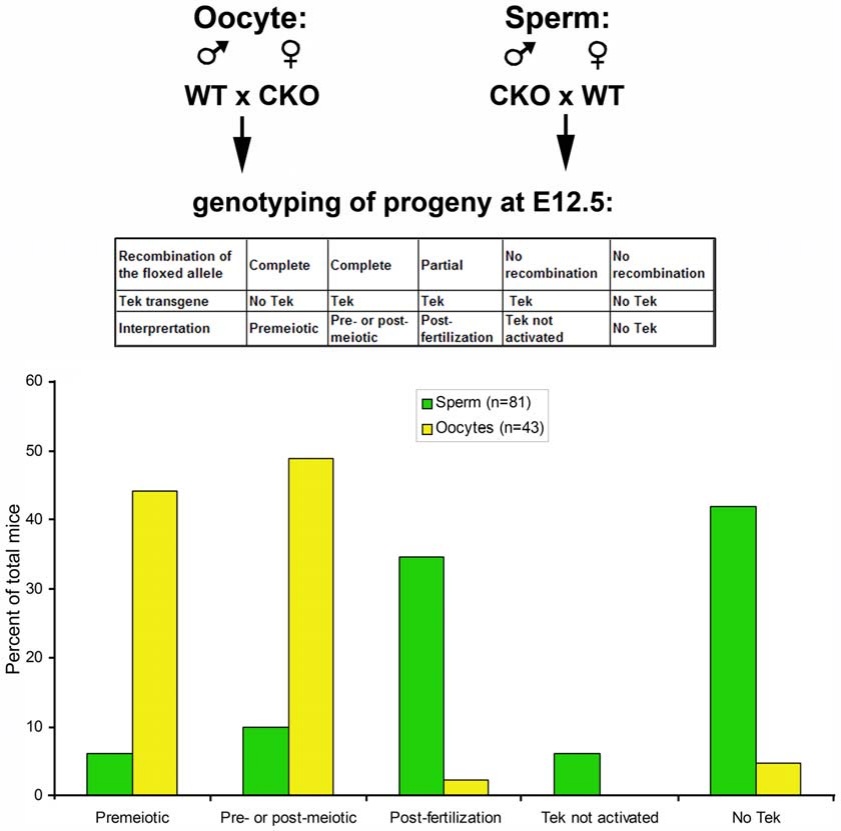
Tek-Cre–dependent recombination rates in male and female germ line. To detect recombination in sperm and oocytes, CKO males and females, respectively, were mated to WT mice, followed by euthanasia of pregnant females and genotyping of the embryos for the Tek transgene and Cre-induced recombination of the floxed allele. Top diagram represents the mating schemes and the genotyping combinations used to assign the categories on the chart shown at the bottom. See also the description in the text.

To address this possibility and to find out how frequently the germ line deletion of Ate1 happens in CKO males and females before and after meiosis, we analyzed the rates of the deletion of Ate1 in female and male germ line by mating CKO and WT males and females in reciprocal combinations (CKO male to WT female and vice versa) and genotyping the fetuses from the resulting matings, collected at E12.5 and E13.5 (Figure 5). These fetuses have one Ate1-floxed allele from the CKO parent and one wild type allele from the control parent. By testing the recombination in these embryos, it can be determined whether this recombination happened in germ cells or in the embryos post-fertilization, as well as those cases where the presence of Tek transgene did not result in recombination (Tek not activated). The following five possibilities were scored, by genotyping the progeny of the CKO × WT matings, as shown in Figure 5: (1) premeiotic deletion: Ate1 floxed-deleted allele (Ate1Δ) without Tek-Cre; (2) pre- or post-meiotic deletion: Ate1Δ allele with Tek-Cre; (3) post-fertilization: both Ate1Δ and intact Ate1-floxed allele with Tek-Cre; (4) Tek not activated: intact Ate1-floxed allele with Tek-Cre; and (5) no Tek: intact Ate1-floxed allele without Tek-Cre.

When CKO females were mated with WT males to test the recombination rates in the oocytes, 44% of the deletion (19 out of 43 analyzed embryos) was premeiotic, and 49% of the deletion (21 out of 43) was pre- or post-meiotic. Since the number of oocytes with and without Tek-cre are expected to be the same, and Tek promoter is expected to be activated at a specific developmental stage, it appears highly likely that both groups represented premeiotic deletion of Ate1 in the oocytes, suggesting that Ate1 is deleted in ∼93.0% of premeiotic germ cells in female CKO mice. In contrast, when CKO males were mated with WT females to determine recombination rates in the male germ line, only 18.1% of male germ cells (13 out of 81 embryos) had deletion before meiosis.

These results suggest that CKO germ cells undergo Ate1 deletion before meiosis, likely resulting in the production of ‘defective’ gametes that cannot support normal development.

## Discussion

**Figure 6:**
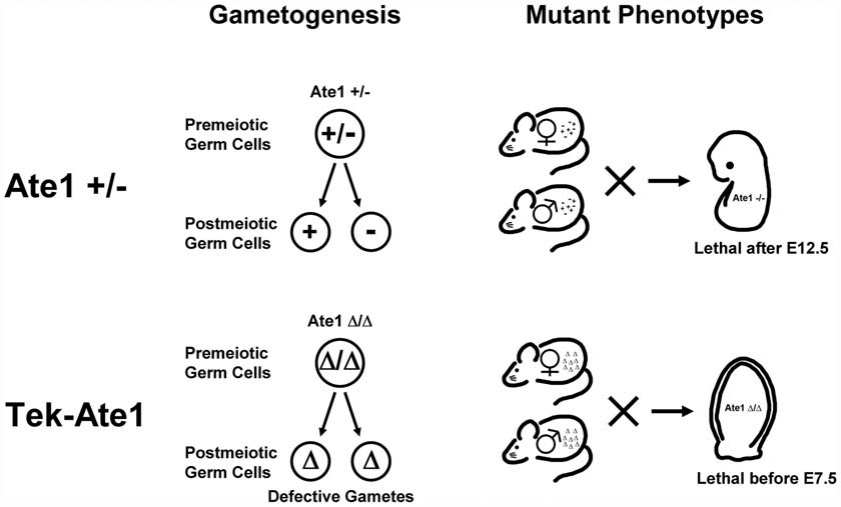
Defects in gametogenesis in Tek-Ate1 lead to early embryonic lethality. Top, in complete Ate1 knockout, gametes are generated from Ate1 +/− germ cells, leading to the formation of normal sperm and oocytes that carry Ate1+ and Ate1- alleles. The combination of Ate1- sperm and oocyte results in embryonic lethality at or after E12.5 as previously described [4]. Bottom, in Tek-Ate1 conditional knockout, Ate1 deletion happens before meiosis, resulting in the impaired gametogenesis and the formation of abnormal Ate1Δ gametes. Such gametes cannot support normal development, and in homozygous combination induce early embryonic lethality after implantation but before E7.5.

In this study, we used a Tek-driven conditional mouse knockout of Ate1 to show that Ate1-mediated protein arginylation plays a previously unknown role in germ cell development. Our results suggest that Tek-Cre-induced deletion of Ate1 in premeiotic germ cells affects gametogenesis, resulting in the formation of defective gametes that cannot support normal embryogenesis. Unlike the complete Ate1 knockout mice that die on or after E12.5 [4], a significant percentage of Tek-Ate1 embryos die at early post-implantation stages, before E7.5, likely due to the defects in gamete formation from Ate1 knockout premeiotic germ cells (Figure 6). These defects are partially masked by the early lethality observed in the commercial Tek-Cre mouse line; according to our data all animals carrying homozygous Tek transgene die early in development, providing a ‘background’ lethality rate of approximately 25%. Since expression of Cre recombinase does not result in lethality in other conditional mouse strains, and since the Jackson Laboratory currently has another Tek-Cre mouse strain in development, which is stated to be viable as Tek homozygotes, we suggest that this lethality originates from the site of insertion of the Tek-Cre transgene, which may disrupt the function of an essential gene.

While early lethality and embryo deterioration precluded us from reliable DNA isolation from the deceased embryos and thus provided an incomplete data on embryo genotypes, our finding that the deletion of Ate1-floxed allele was observed in 93% of oocytes and 18% of sperm (Figure 5) allowed us to calculate probabilities of the genotypes in the offspring of CKO × CKO matings (Table 1; see Table S1 and Table S2 for detail). Homozygous Tek-Cre is expected to occur with 25% frequency and result in complete lethality before E7.5, however, in CKO × CKO matings more than 40% of embryos die at this stage, suggesting that the remaining ∼15% on top of the Tek-Cre-induced lethality is due to the deletion of the Ate1-floxed allele (denoted Δ in the tables and in Figure 6). Based on these numbers and on the expectation that homozygous Ate1 deletion should be more severe than others, it appears likely that Ate1 Δ/Δ genotype, expected to occur with 12.6% frequency, results in embryonic lethality before E7.5, bringing the combined lethality from Tek homozygous and Ate1Δ/Δ to ∼37.6% -- the number similar to the 40% lethality seen in our experiments (Figure 4).

**Table 1:** Expected genotypes in embryos from CKO × CKO mating.

Tek-cre	Ate1 ♀/♂	Probability (%)
Homozygous	Any	25.0
Heterozygous or none	Δ/Δ	12.6
Heterozygous	Δ/f	38.1
None	Δ/f	19.1
Heterozygous	f/Δ	0.6
None	f/Δ	0.3
Heterozygous	f/f	2.9
None	f/f	1.4
**Total**		**100.0**

When Tek-Ate1 females were mated with Ate1 +/− males, 46.5% of embryos were expected to be Ate1 Δ/−, yet the early (before E7.5) death rate in these matings was only 8.1%, suggesting that the Ate1 Δ/− mice, which lack both Ate1 alleles, can survive at least until E7.5 (Figure 4 and Table S2). Such mice likely die after E12.5, similarly to complete Ate1 knockout.

Since Ate1 Δ/Δ mice die much earlier in development than the previously characterized Ate1−/− mice, it is clear that the Ate1 Δ/Δ phenotype is much more severe than Ate1 Δ/− or Ate1 −/−. The only plausible explanation for this difference is that premeiotic Ate1 deletion in the Ate1 Δ/Δmice affects gametogenesis, resulting in the formation of severely defective gametes that cannot support development past implantation. Indeed, in Ate1−/− mice the gametes carrying the knockout allele develop from Ate1+/− precursors, which contain a functional arginyltransferase and have never been found to have any defects. In agreement with this, the first generation CKO progeny of Tek floxed/+ mice, whose mothers carry the post-meiotic Ate1 Δ allele, is viable (Figure 1A).

Combined lethality of Tek homozygous and Ate1 Δ/Δ mice amounts to ∼40%, yet the lethality at term seen in our experiments is much higher (estimated lethality rate over 80%, see Figure 1), suggesting that a significant number of embryos (40% or more) develop normally past E8.5 but die before birth. Based on the expected genotypes shown in Table 1, it appears likely that these embryos have Ate1 Δ/f genotype, i.e., develop from one defective gamete affected by premeiotic Ate1 deletion and one normal floxed allele. It is possible that in such embryos the effect of the defective Ate1 Δ gamete is combined with the effect of Tek-Cre-driven later-stage deletion of Ate1 in endothelial and/or hematopoetic cells. According to our calculations (Table 1), an estimated 57% of embryos carry the Ate1 Δ/f genotype. If all these embryos could not survive until birth, the rates of lethality in CKO matings would have been close to 100%. Therefore, it appears likely that the developmental impact of one defective gamete is variable and some of these embryos can still develop to term.

It has been previously reported that Tek-Cre is active in the female germ line, but not in the male germ line [12], however the Jackson Laboratory reports that a small percentage of the male germ line is also expressing the Tek-Cre transgene. In our study 18% of fetuses had Ate1 Δ allele of paternal origin (Figure 5). While some of this deletion could originate from the mothers of CKO male mice, it appears likely that at least some of these cases originate from partial Tek-Cre expression in the male germ line. In our study we have addressed the possibility of CKO-related chromosomal defects in the germ cells in the testes and found that these cells have normal karyotypes (Figure S3).

In summary, our data show that embryos from CKO matings that are homozygous for Ate1 Δ allele die before E7.5, much earlier than regular Ate1 KO (Ate1 −/−), suggesting that Ate1 Δ gametes have severe defects and cannot support normal development (Figure 6). In females, premeiotic Ate1 deletion results in the formation of defective oocytes. In males, depending on whether Tek-cre is activated in the germ line, the deletion of Ate1 allele in the sperm happens either before or during meiosis. In the case of premeiotic deletion, the resulting sperm is expected to have defects similar to those in oocytes. In the case of postmeiotic deletion, such sperm would behave similarly to regular Ate1 knockout. It is possible that germ cell development in the CKO males is affected by Tek-Cre-driven Ate1 deletion in endothelial and/or hematopoietic cells.

In addition, large part of embryos heterozygous for Ate1Δ die after E12.5, suggesting that the defects in Ate1Δ gametes cannot be completely rescued by an intact floxed allele, and pointing to a possibility that in such embryos Tek-Cre-driven Ate1 deletion in other tissues may contribute to lethality.

Deletion of Ate1 in premeiotic germ cells could result in significant changes in gametogenesis, leading to the high rates of embryonic lethality seen in our study. Indeed, Ate1 function has been implicated in the regulation of the cytoskeleton and a large subset of identified arginyltransferase substrates (including actin, tubulin, and dynein [6]) play key roles in cell division, meiotic chromosome segregation and sperm motility (see [13]–[17] for overview). Impaired arginylation of these proteins at premeiotic stages could cause disregulation of meiosis, leading to abnormal nuclear shaping, impaired cell communication, aneuploidy of oocytes or sperm, and/or abnormal distribution of the essential components in the oocyte cytoplasm. While such germ cells may be able to undergo normal fertilization, the effect of such disregulation, in combination with continuing abnormalities during cell division, would lead to developmental delay, embryo abnormalities, and death at early post-implantation stages as seen in our study. Since identification of proteins arginylated in vivo is only at its initial stages, it is also possible that some of the arginylated proteins, or proteins regulated by arginyltransferase, have additional roles in germ cell development, for example in regulating the epigenetic modifications essential for normal gametogenesis. Studies of the underlying mechanisms that link premeiotic Ate1 function with normal development using germ line-specific Ate1 knockout mouse models constitute an exciting direction of further research.

## Materials and Methods

### Mouse strains used in this study

For different experiments in this study, the following mouse strains were used: B6.Cg-Tg(Tek-cre)12Flv/J (Tek-Cre, The Jackson Laboratory, stock number 004128); Ate1-floxed and Tek-Ate1 conditional knockout mice (CKO) (see below for the mouse strain generation); Ate1 +/− [5].

Animals were maintained and used for the experiments according to the guidelines of the Institutional Animal Care and Use Committee (IACUC) of the University of Pennsylvania.

### Generation of Ate1-floxed and Tek-Ate1 mice

Mice with the exons 1-3 of the Ate1 gene flanked by LoxP sites (Ate1-floxed mice) were generated by introducing a targeting construct into the corresponding genome region in a cassette containing the floxed allele of the Ate1 genomic region fused with an frt-flanked Neo gene. The targeting vector was constructed using recombineering technique as described in [18]. A 12,375 bp genomic DNA fragment (position Chr 7: 130,302,694 - 130,315,068 Mouse Feb 2006 Assembly) containing exon 1–4 of the gene was retrieved from BAC clone RP23-92D13. A loxP sequence was inserted 657 bp upstream of exon 1 and a frt-neo-frt-loxP cassette was inserted into the intron 3, 505 bp downstream of exon 3. Thus a fragment of 5,019 bp genomic DNA containing exons 1–3 of the Ate1 gene was floxed (see Figure S1A). For ES cell targeting, the targeting vector was linearized with Not1 and electroporated into D1 ES cells derived from F1 hybrid blastocysts of 129S6 × C57BL/6J by Gene Targeting & Transgenic Facility at University of Connecticut Health Center. 192 G418 resistant ES colonies were isolated, and 32 clones were screened by nested PCR using primers outside the construct paired with primers inside the neo cassette. The sequences for primers used for ES cell screening were as follows:

5′ arm forward primers: ATE Scr F1 (5′- GTCTCACTTCCCTTCCTTAG -3′) and ATE Scr F2 (5′- ATTACCAGTGCTCGGTGCTT -3′). Reverse primers: LoxP scrR1 (5′–GAGGGACCTAATAACTTCGT-3′) and LoxP scrR2 (5′-GGAATTGGGCTGCAGGAATT-3′). 3′ arm forward primers: frt scr F1 (5′-TTCTGAGGCGGAAAGAACCA-3′) and frt scr F2, (5′-CGAAGTTATTAGGTGGATCC-3′); Reverse primers: ATE Scr R1 (5′- TCAGTGGTTCTCAACCTGTG -3′) and ATE Scr R2 (5′- CAGGGGTTACCTAAGACCAT -3′);

7 out of 32 clones were PCR positive for both arms and were expanded. The genotypes were confirmed after ES cell expansion.

For chimera generation and F1 mice genotype analysis three clones (1B3, 1A4 and 1H2) were aggregated with 8-cell embryos of CD-1 strain. The aggregated embryos were transferred to pseudopregnant recipients and allowed to develop to term. 25 chimeric mice were identified by coat color. Five chimeras (2 each for1B3 and 1A4, 1 for 1H2) were mated with CD-1 females to test for germ line transmission. All of them were demonstrated as germ line chimeras. The neo cassette was removed by mating the chimeras with ROSA26FLP1 (Jax stock#: 003946) homozygous females.

To obtain Tek-Ate1 (CKO) mice, Ate1-floxed mice were crossed with Tek-Cre strain expressing Cre recombinase under Tek, endothelial-specific receptor tyrosine kinase (also known as Tie2), promoter. For LacZ reporter experiments shown in the Figure S2 Tek-Cre mice were crossed with the commercial ROSA reporter mouse strain B6.129S4-Gt(ROSA)26Sortm1Sor/J (The Jackson Laboratories).

The following primers were used for genotyping of the final mouse strain (see Figure S1 for a typical genotyping gel): for Ate1-floxed allele, Ate1gtLoxF (5′-TGCCTCCAGCATTGGATGAA-3′) and Ate1gtLoxR (5′-CCATGGGTCTCCAATTTGCA-3′); for complete and partial Tek-Cre-induced recombination, Ate1gtLoxF and Ate1gtLoxR were used in combination with a third primer, ATE1gt frtR2 5′-AGACAGGGCCTCATCAAGTA-3′

For Tek transgene, primers recommended by the Jackson Laboratory were used as described on their web site.

Fetuses used in this study were produced by natural mating or embryo transfer (see below).

### Embryo transfer

Superovulation and embryo transfer protocol were carried out as described in [19]. In brief, female mice, superovulated by injection with Pregnant Mare Serum Gonadotropin (PMSG) followed after 48 hrs by an injection with human chorionic gonadotropin (hCG), were mated with male mice. Mated females which had copulation plugs in the morning after mating (0.5 day post coitum (dpc)) were euthanized by CO2 administration followed by cervical dislocation at 2.5 dpc. Both left and right oviducts were flushed with M2 medium (Millipore, MR-015P-5D) to recover the embryos at embryonic day 2.5 (E2.5). Recovered embryos were transferred into the oviducts of pseudopregnant females at 0.5 dpc that had been mated with vasectomized ICR male mice.

### Collection of fetuses, deciduas, and yolk sacs

Fetuses and yolk sacs were collected at the stages between E8.5 and E13.5. The fetuses and deciduas were evaluated and photographed under dissecting microscopes, and DNA was extracted from yolk sacs and/or embryo tissues for genotyping.

### Histology of uterine horns

The uterine horns at 7.5dpc and 8.5dpc were collected from pregnant female mice euthanized as described above, and immediately fixed in 4% paraformaldehyde in PBS at 4°C. Fixed samples were washed in PBS, embedded in paraffin, and sectioned. Slide sections were stained with hematoxylin and eosin.

### Analysis of preimplantation embryos

Embryos from the females superovulated and mated as described above were collected at E2.5 and cultured for one day in KSOM media (Millipore) under 5% CO_2_ at 37°C. Embryos at 4–8 cell stage, morulae, and blastocysts were counted manually from the images photographed at 10x and 40x and derived as percentages for control (Ate1-floxed) or CKO mice as described in the text and shown in Figure 2D.

### Analysis of sperm- and oocyte-specific recombination

To analyze sperm- and oocyte-specific recombination, CKO males and females were mated to wild type mice in reciprocal combinations (CKO male to WT female and vice versa). In each mating, females were euthanized at dpc12.5 or 13.5, embryos were collected and genotyped for the Tek transgene and the recombined floxed allele. Recombination events were scored as shown in Figure 5, top and as described in the text.

### Karyotyping

Karyotyping of male germ cells was carried out as described in [20].

## Supporting Information

Figure S1 Generation of Tek-Ate1 conditional knockout mouse.(A) Diagram showing the general strategy for the construction of Ate1-floxed mouse line and Tek-Cre-driven excision of the Ate1-floxed allele. (B) typical genotyping gels for Ate1-floxed allele (left), Tek-Cre transgene (middle), and Cre-driven recombination (right). In the floxed genotyping, detection of 358 bp and 283 bp bands served as evidence of the presence of the floxed and wild-type allele, respectively. In Tek genotyping, the transgene was detected by the presence of a 100 bp band. In the genotyping for the floxed allele recombination, in addition to the wild type and floxed allele as described above, detection of an additional product of 220 bp served as evidence of the recombination of the floxed allele. Genotypes were assigned as ‘partial’ or ‘complete’ based on the presence or absence of the floxed allele together with the recombined band. Only the heterozygous animals (derived from the floxed/+ genotype) are shown in the figure.
(64.70 MB TIF)

Figure S2 Tek-Cre expression occurs at high level in the knockout embryos. Tek-Cre mice were crossed with R26R Rosa reporter strain and stained with X-gal to visualize lacZ. A litter at E10.5 is shown.
(32.12 MB TIF)

Figure S3 CKO germ cell precursors in the testis have normal chromosome numbers. Left, chromosome spreads from the control and CKO testes stained with Giemsa. Right, quantification of chromosome numbers in control and CKO spreads show no abnormalities in the CKO chromosome count.
(61.10 MB TIF)

Table S1 Expected genotypes in gametes and embryos in CKO × CKO mating.
(0.06 MB DOC)

Table S2 Expected genotypes in gametes and embryos in CKO × Ate1 +/− mating.
(0.04 MB DOC)
