# Supplementary figures and images for: Conditional Tek Promoter-Driven Deletion of Arginyltransferase in the Germ Line Causes Defects in Gametogenesis and Early Embryonic Lethality in Mice
Source: PLoS One. 2009 Nov 5;4(11):e7734. doi: 10.1371/journal.pone.0007734 (PMC2767504; doi:10.1371/journal.pone.0007734)

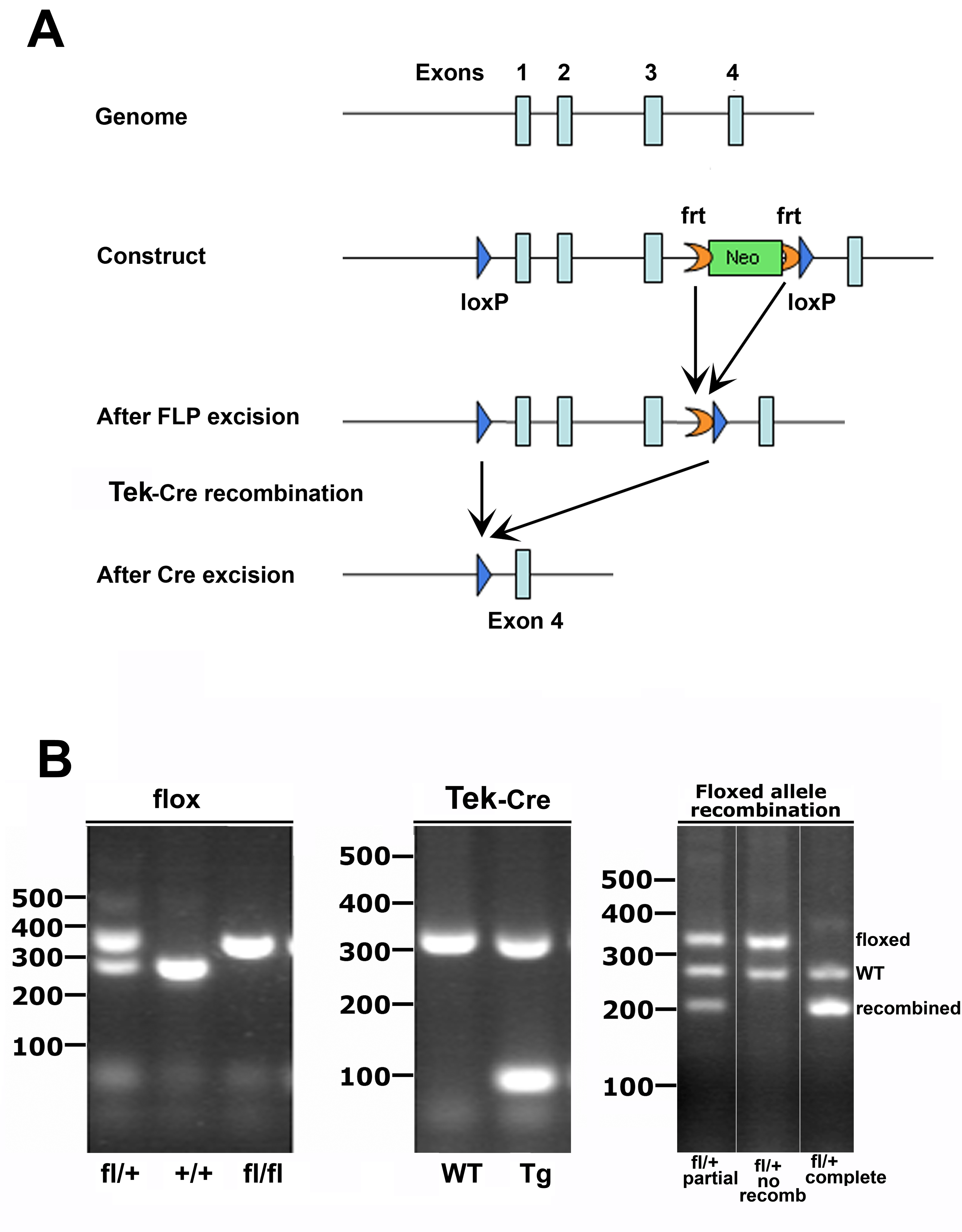

Supplement: Figure S1 — Generation of Tek-Ate1 conditional knockout mouse.(A) Diagram showing the general strategy for the construction of Ate1-floxed mouse line and Tek-Cre-driven excision of the Ate1-floxed allele. (B) typical genotyping gels for Ate1-floxed allele (left), Tek-Cre transgene (middle), and Cre-driven recombination (right). In the floxed genotyping, detection of 358 bp and 283 bp bands served as evidence of the presence of the floxed and wild-type allele, respectively. In Tek genotyping, the transgene was detected by the presence of a 100 bp band. In the genotyping for the floxed allele recombination, in addition to the wild type and floxed allele as described above, detection of an additional product of 220 bp served as evidence of the recombination of the floxed allele. Genotypes were assigned as ‘partial’ or ‘complete’ based on the presence or absence of the floxed allele together with the recombined band. Only the heterozygous animals (derived from the floxed/+ genotype) are shown in the figure. (64.70 MB TIF) [file pone.0007734.s001.tif]

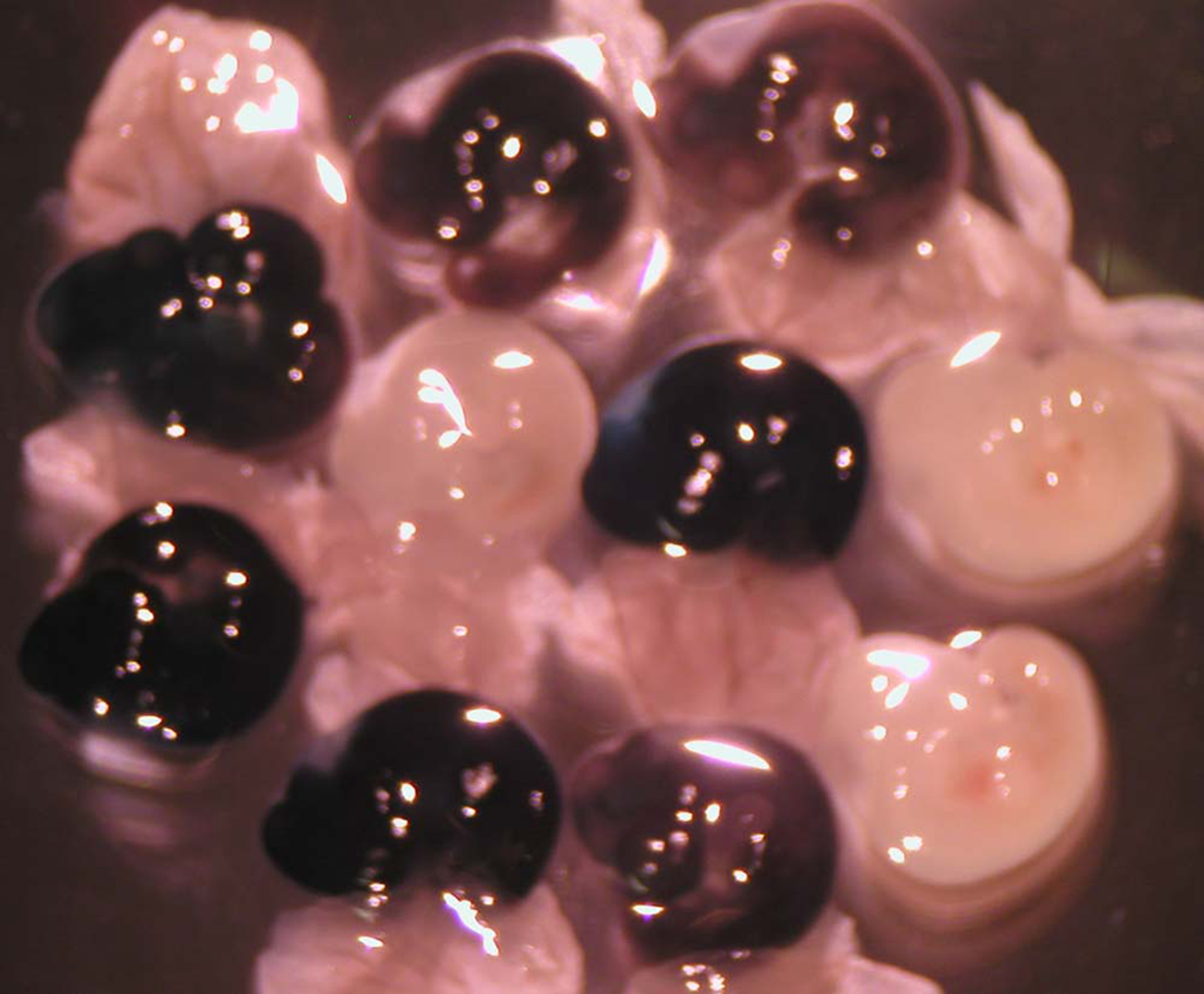

Supplement: Figure S2 — Tek-Cre expression occurs at high level in the knockout embryos. Tek-Cre mice were crossed with R26R Rosa reporter strain and stained with X-gal to visualize lacZ. A litter at E10.5 is shown. (32.12 MB TIF) [file pone.0007734.s002.tif]

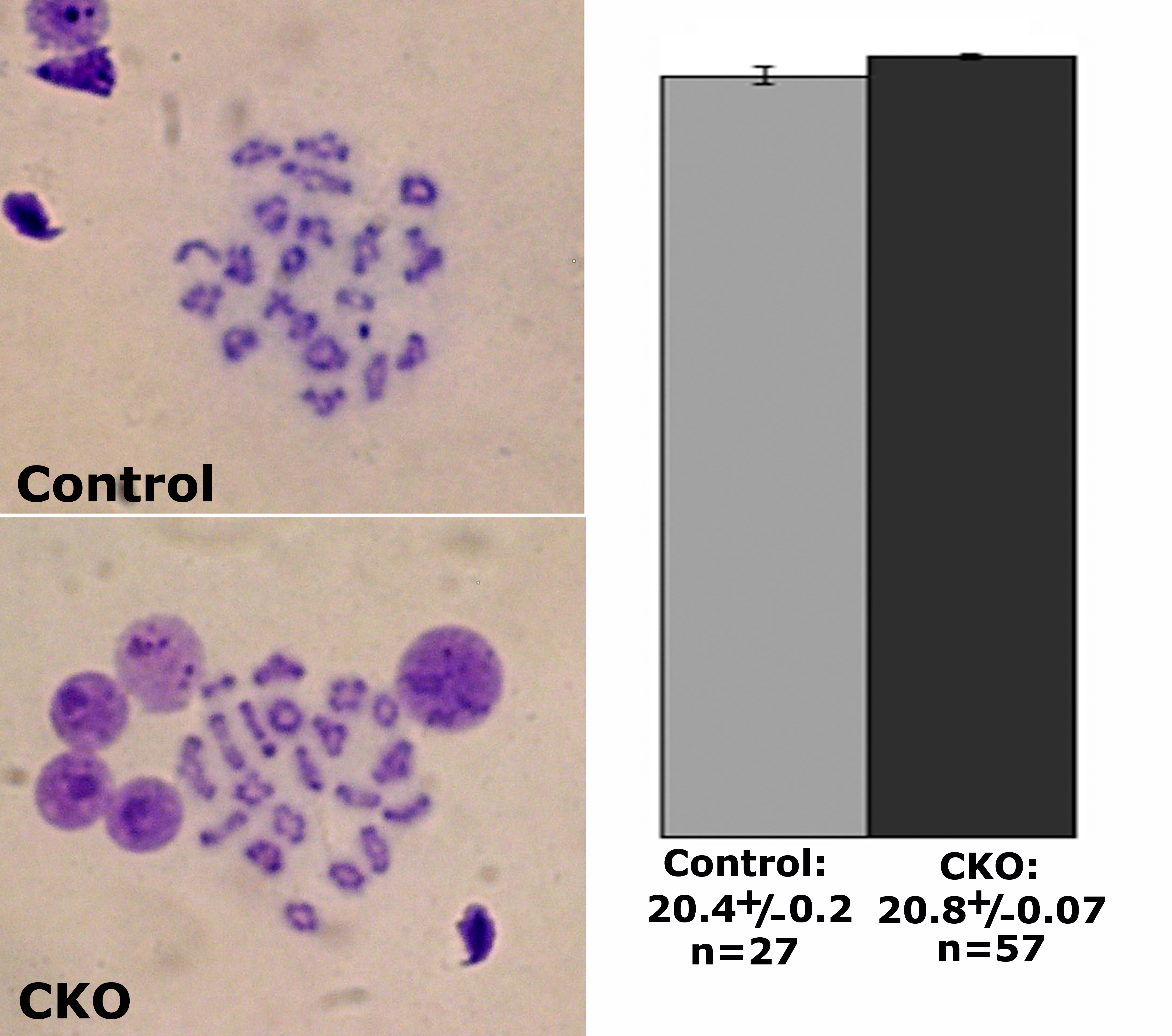

Supplement: Figure S3 — CKO germ cell precursors in the testis have normal chromosome numbers. Left, chromosome spreads from the control and CKO testes stained with Giemsa. Right, quantification of chromosome numbers in control and CKO spreads show no abnormalities in the CKO chromosome count. (61.10 MB TIF) [file pone.0007734.s003.tif]
