# Supplementary material for: Conditional Tek Promoter-Driven Deletion of Arginyltransferase in the Germ Line Causes Defects in Gametogenesis and Early Embryonic Lethality in Mice
Source: PLoS One. 2009 Nov 5;4(11):e7734. doi: 10.1371/journal.pone.0007734 (PMC2767504; doi:10.1371/journal.pone.0007734)
Supplement: Table S1 — Expected genotypes in gametes and embryos in CKO × CKO mating. (0.06 MB DOC) [file pone.0007734.s004.doc]

| **Supplemental Table 1. Expected genotypes in gametes and embryos in CKO x CKO mating** | | | | | | | | | | |
| --- | --- | --- | --- | --- | --- | --- | --- | --- | --- | --- |
| CKO Female | | | X | CKO Male | | |  | Embryos | | |
| Tek-Cre | Ate1 | Probability |  | Tek-Cre | Ate1 | Probability |  | Tek-Cre | Ate1 ♀/♂ | Probability |
| + | Δ | 0.465 |  | + | Δ | 0.09 |  | Homozygous | Δ/Δ | 4.185% |
| + | Δ | 0.465 |  | - | Δ | 0.09 |  | Heterozygous | Δ/Δ | 4.185% |
| + | Δ | 0.465 |  | + | f | 0.41 |  | Homozygous | Δ/f | 19.065% |
| + | Δ | 0.465 |  | - | f | 0.41 |  | Heterozygous | Δ/f | 19.065% |
| - | Δ | 0.465 |  | + | Δ | 0.09 |  | Heterozygous | Δ/Δ | 4.185% |
| - | Δ | 0.465 |  | - | Δ | 0.09 |  | None | Δ/Δ | 4.185% |
| - | Δ | 0.465 |  | + | f | 0.41 |  | Heterozygous | Δ/f | 19.065% |
| - | Δ | 0.465 |  | - | f | 0.41 |  | None | Δ/f | 19.065% |
| + | f | 0.035 |  | + | Δ | 0.09 |  | Homozygous | f/Δ | 0.315% |
| + | f | 0.035 |  | - | Δ | 0.09 |  | Heterozygous | f/Δ | 0.315% |
| + | f | 0.035 |  | + | f | 0.41 |  | Homozygous | f/f | 1.435% |
| + | f | 0.035 |  | - | f | 0.41 |  | Heterozygous | f/f | 1.435% |
| - | f | 0.035 |  | + | Δ | 0.09 |  | Heterozygous | f/Δ | 0.315% |
| - | f | 0.035 |  | - | Δ | 0.09 |  | None | f/Δ | 0.315% |
| - | f | 0.035 |  | + | f | 0.41 |  | Heterozygous | f/f | 1.435% |
| - | f | 0.035 |  | - | f | 0.41 |  | None | f/f | 1.435% |
|  |  |  |  |  |  |  |  | Total | | 100.00% |
