# Supplementary material for: Conditional Tek Promoter-Driven Deletion of Arginyltransferase in the Germ Line Causes Defects in Gametogenesis and Early Embryonic Lethality in Mice
Source: PLoS One. 2009 Nov 5;4(11):e7734. doi: 10.1371/journal.pone.0007734 (PMC2767504; doi:10.1371/journal.pone.0007734)
Supplement: Table S2 — Expected genotypes in gametes and embryos in CKO × Ate1 +/− mating. (0.04 MB DOC) [file pone.0007734.s005.doc]

| **Supplemental Table 2. Expected genotypes in gametes and embryos in CKO x Ate1 +/- mating** | | | | | | | | | | |
| --- | --- | --- | --- | --- | --- | --- | --- | --- | --- | --- |
| CKO Female | | | X | Ate1 +/- Male | | |  | Resulting Fetus | | |
| Tek-Cre | Ate1 | Probability |  | Tek-Cre | Ate1 | Probability |  | Tek-Cre | Ate1 ♀/♂ | Probability |
| + | Δ | 0.465 |  | - | + | 0.5 |  | Heterozygous | Δ/+ | 23.25% |
| + | Δ | 0.465 |  | - | - | 0.5 |  | Heterozygous | Δ/- | 23.25% |
| - | Δ | 0.465 |  | - | + | 0.5 |  | None | Δ/+ | 23.25% |
| - | Δ | 0.465 |  | - | - | 0.5 |  | None | Δ/- | 23.25% |
| + | f | 0.035 |  | - | + | 0.5 |  | Heterozygous | f/+ | 1.75% |
| + | f | 0.035 |  | - | - | 0.5 |  | Heterozygous | f/- | 1.75% |
| - | f | 0.035 |  | - | + | 0.5 |  | None | f/+ | 1.75% |
| - | f | 0.035 |  | - | - | 0.5 |  | None | f/- | 1.75% |
|  |  |  |  |  |  |  |  | Total | | 100.00% |
